# Knowledge and preventive practices associated with anticipatory action for climate-related cholera outbreaks in Mozambique: a gender perspective

**DOI:** 10.21203/rs.3.rs-10341621/v1

**Published:** 2026-08-12

**Authors:** Taimo Taimo, Noa Beca, Luís Artur

**Affiliations:** 1,2,3 Department of Economy and Agrarian Development, Faculty of Agronomy and Forestry Engineering, Eduardo Mondlane University, Julius Nyerere Avenue, n. 3453, Maputo 1102, Mozambique

**Keywords:** Cholera, Gender, KAP, Anticipatory Action

## Abstract

**Background:**

Cholera remains a major public health concern in sub-Saharan Africa, exacerbated by extreme climatic events and inadequate WASH-related practices. As both climate change impacts and WASH practices are gender-sensitive, this study adopts a gendered perspective to examine the relationship between anticipatory action (AA) for climate-induced cholera outbreaks and knowledge, attitudes and practices (KAP) in Zambézia Province, Mozambique.

**Methods:**

A cross-sectional survey was conducted in Quelimane (urban) and Mopeia (rural) using a multistage sampling design. A total of 448 respondents were interviewed. Data were analyzed using descriptive statistics and gender-based comparisons. Negative binomial regression was applied to assess associations between KAP, gender and AA.

**Results:**

The findings revealed sociodemographic inequalities between men and women, with women being younger and more frequently widowed. A critical KAP gap was observed, with very low levels of cholera knowledge (96.2% low) and insufficient practices (86.6% low), despite generally positive attitudes toward disease prevention (76.3% high). Anticipatory readiness was extremely limited, with 96.8% of respondents classified as having low preparedness levels. In the negative binomial model, knowledge (IRR = 1.069), practices (IRR = 1.072), and age between 36 and 60 years (IRR = 1.106) were significantly associated with the expected number of AA.

**Conclusions:**

Cholera preparedness remains limited despite high disease awareness and generally positive attitudes. Knowledge and preventive practices were the main factors associated with the adoption of AA, whereas attitudes and gender were not significantly associated in the adjusted model. These results underscore the need for integrated interventions that combine health education, strengthened WASH conditions, early warning mechanisms and gender-sensitive strategies.

## 1 Introduction

Cholera is an acute diarrheal disease caused by the bacterium *Vibrio cholerae*, transmitted primarily through the ingestion of contaminated water or food, and it remains a major public health problem, particularly in sub-Saharan Africa [1–2]. Scientific evidence shows that its occurrence and spread are strongly associated with environmental and structural determinants, such as limited Water, Sanitation and Hygiene (WASH) access to safe drinking water, inadequate sanitation, and poor hygiene conditions [3–4]. These factors collectively facilitate the persistence of transmission, especially in vulnerable settings.

Climate change is an increasingly recognized as a major driver of cholera risk. Climatic drivers such as heavy rainfall, rising temperatures, and extreme events (cyclones and floods) significantly magnify the incidence and seasonality of cholera by compromising WASH infrastructure and increasing the contamination of water sources [5–6].

In Mozambique, cholera is endemic, with recurrent outbreaks associated with both seasonal patterns and extreme climatic events [7–8]. Epidemiological surveillance data indicate that the country experiences cholera episodes almost annually, with a higher concentration in the central and northern regions [7]. Annual incidence ranges from 0 to 211 cases per 100.000 inhabitants, with case fatality rates between 0.2% and 4.3% [8]. Between September 2022 and April 2023, the country experienced one of its largest recent outbreaks, with more than 43.000 cases and over 160 deaths, mostly in provinces of Zambézia, Nampula, and Sofala [9].

In this context, anticipatory action (AA) has emerged as a promising approach to reducing the outbreaks and potential disastrous impacts. AA refers to the implementation of pre-planned, ex-ante financed interventions triggered by early systems signals, with the aim of mitigating adverse effects before the occurrence or escalation of an event [10]. Initially applied to extreme weather events, this approach has been progressively adapted to the management of climate-sensitive disease outbreaks, such as cholera [10–11].

However, the effectiveness of AA also depends on how social vulnerabilities are recognized and incorporated into the design of responses. Evidence shows that women, who are often responsible for water management and household healthcare, face greater exposure to risks despite playing a central role in prevention [12–13]. In addition, structural and sociocultural barriers may limit women’s access to information, resources, and decision-making processes, thereby reducing the effectiveness of preventive and response interventions. In Cabo Delgado, Mozambique, significant differences between men and women regarding cholera-related knowledge and preventive practices have been observed, reinforcing the importance of integrating a gender perspective into risk analysis [14].

In this sense, the literature on AA highlights that the early implementation of measures such as the distribution of WASH kits, the promotion of hygiene practices, and other early interventions has the potential to significantly reduce cholera transmission, particularly when triggered by epidemiological or climatic indicators. It is also essential to integrate gender-disaggregated data into the design, implementation, and monitoring of responses in order to enhance their effectiveness [10]. Gender also influences people’s knowledge, attitudes, and practices (KAP) toward diseases. Given the fact that women and men have differentiated access to resources and have been socially assigned different roles and spaces, their KAP might differ considerably with implications on how they prepare, respond and recover from diseases [15–16].

Despite that recognition of gendered KAP towards diseases, important gaps persist in the systematic incorporation of gender-disaggregated KAP analyses. Many studies have rightly pointed out the relevance of KAP in framing health intervention [14,17–20]. However they have missed a gender dimension and have been carried out of the AA scope. This limits effective, gender sensitive AA and the development of truly inclusive preparedness and responses that are sensitive to differentiated vulnerabilities.

To address these gaps, we developed a conceptual framework that integrates evidence from the literature and guides the analytical approach adopted in this study (Figure 1). The framework is based on the premise that the occurrence of cholera outbreaks results from the interaction between environmental, structural, and behavioral determinants [3]. Extreme climate events, such as cyclones and floods, degrade water and sanitation infrastructure, increase environmental contamination, and elevate population exposure to the fecal–oral transmission of *Vibrio cholerae* [5].

In contexts such as Mozambique, where outbreaks are recurrent and spatially concentrated, vulnerability is exacerbated by persistent limitations in access to safe water and sanitation, particularly in areas affected by population displacement and the destruction of basic services [7,21–22].

Within this process, anticipatory action acts as a preventive mechanism by mobilizing interventions before the onset of an outbreak or prior to an escalation in risk. Anticipatory action includes triggers based on climatic, epidemiological, or operational alerts, enabling early measures such as risk communication, distribution of WASH kits, strengthened surveillance, pre-positioning of supplies, and, when applicable, vaccination or logistical support [10]. Recent studies show that AA can be integrated into cholera decision-making and supported by specific models and analytical frameworks [23].

The gender dimension cuts across the entire process, as women and men may differ in exposure, access to information, caregiving burden, decision-making autonomy, and the ability to adopt preventive measures [13]. In contexts of displacement and water scarcity, women tend to bear greater domestic and sanitation responsibilities, and the literature also shows that factors such as education, phone ownership, and the presence of soap in the household are associated with better cholera-related knowledge, attitudes, and practices [12,14]. Thus, gender is conceptualized in this study as an explanatory variable whose effects on AA are potentially mediated by KAP components.

Based on this, the conceptual framework presented in Figure 1 proposes that AA and KAP influence community preparedness and response to cholera, while gender shapes how these responses are developed and implemented.

In this context, the present study aims to examine gender differences in knowledge, attitudes, and practices (KAP) and their association with anticipatory action (AA) in the context of cholera outbreaks in Zambézia Province, Mozambique. The study seeks to contribute to the development of more gender-sensitive and effective AA intervention strategies for reducing the burden of the disease.

## 2 Methods

### 2.1 Research Site

Research was carried out in Quelimane and Mopeia districts in Zambezia province (Figure 2). Zambézia stands as the poorest province in Mozambique with an average monthly per capita consumption expenditure of approximately USD 13 [24]. It also ranks as the most vulnerable province to waterborne diseases (WBD) having reported more cases over the past 10 years than any other province; 16 out of its 22 districts, that is 72.7 percent of the province (the highest level across the country) are mapped as vulnerable to WBD [25]. Notably Quelimane and Mopeia have reported WBD every year, driven primarily by recurring annual floods. In both areas livelihoods rely on agriculture, fishing, petty trade, formal and informal employment, remittances, use of forest products and livestock rearing [26–27]. Quelimane is an urban municipality, while Mopeia is a predominantly rural district situated approximately 120 km from Quelimane.

### 2.2 Sampling Strategy

A multi-stage sampling design was employed to select respondents for the study. In the first stage, communities within Quelimane and Mopeia with historically elevated WBD cases accounts were purposively selected. In the second stage, community-level mapping was conducted with the assistance of local leaders and guides to identify settlement patterns and facilitate navigation within each community. The role of local guides was limited to community entry and orientation, they were not involved in household selection. In the third stage, households were selected using a structured random-walk systematic sampling procedure. Enumerators began from a central landmark, typically a local school or village meeting point, and selected a random direction before proceeding through the community. Households were then approached at a fixed interval of every third household along the route, thereby reducing interviewer discretion and potential selection bias. At each selected household, one eligible adult respondent, aged 18 years or older, was interviewed. Eligibility criteria also included informed consent and residence in the area for at least five consecutive years. If a selected household did not meet the inclusion criteria or declined participation, the next eligible household was approached. Overall, this procedure ensured a transparent and systematic respondent selection process while maintaining methodological rigor. It is particularly appropriate in many developing-country contexts, such as sub-Saharan Africa, where household sampling frames are often absent or incomplete [28].

Table 1 presents the distribution of respondents by gender in the districts of Quelimane and Mopeia, highlighting the proportions of men and women in each district, expressed as frequencies (N).

### 2.3 Data Collection and Measures

Quantitative data were collected electronically using Kobo Collect, a mobile data collection platform that allows real-time entry of responses and incorporates validation checks to reduce missing or inconsistent data during interviews. Data collection was conducted in early August 2024, coinciding with the post-harvest dry period where households are less busy with agricultural activities-the main livelihood source. Prior to the main survey, the questionnaire was pre-tested in a comparable community to assess clarity, cultural appropriateness, and the logical sequencing of questions. Feedback from the pretest informed minor revisions to improve wording and ensure the reliability of the survey instrument.

Following these preparations, primary data was collected through structured face-to-face interviews with household heads using a pre-designed questionnaire. The survey captured information on household demographic characteristics, livelihood activities and WASH practices and preparedness. Enumerators conducting the interviews were fluent in Portuguese and the predominant local language “Chuabo” in Quelimane and “Sena” in Mopeia. All researchers adhered to ethical research standards, including voluntary participation, prior informed consent, and the protection of respondent confidentiality.

### 2.4 Model Specification and Estimation Strategy

Factors associated with AA adoption were assessed using a multivariable negative binomial regression model, an approach appropriate for count outcomes exhibiting overdispersion. While, the Poisson model imposes the restrictive assumption of equidispersion, the negative binomial model relaxes this restriction by introducing an additional dispersion parameter, thus the negative binomial specification is preferred when the variance exceeds the mean [29–30].

In this study, the dependent variable was AA_score, treated as a count variable representing the number of anticipatory actions reported by each respondent. The model was estimated using a log link function, allowing the exponentiated coefficients to be interpreted as incidence rate ratios (IRRs). IRR values greater than 1 indicate an increase in the expected number of anticipatory actions, whereas values below 1 indicate a decrease [29–30].

To examine the independent association between anticipatory actions and the explanatory variables, the following covariates were included: Gender, Age, Marital status, Educational level, Household size, Monthly income, Climate-related displacement, Knowledge score, Attitude score, and Practice score. The fully adjusted specification allowed the association of each predictor with the expected number of AA to be estimated while holding the remaining covariates constant (Equation 1).

(Equation 1) $$log\left(\mu_{i}\right)=\beta_{0}+\beta_{1}X_{1i}+\beta_{2}X_{2i}+\cdots +\beta_{k}X_{ki}$$

Where *μ*_*i*_ = *E*(*Y*_*i*_ ∣ *X*_*i*_) represents the expected number of AA for respondent *i*, conditional on the vector of covariates *X*_*i*_. Within this framework, the exponentiated coefficients are interpreted as incidence rate ratios (IRR).

### 2.5 Statistical analysis

All quantitative analyses were conducted using Stata version 17. Descriptive statistics were used to characterize sociodemographic variables and to describe the distribution of KAP scores, as well as AA. Continuous variables were summarized using means and standard deviations, while categorical variables were presented as frequencies and percentages. Differences between genders were statistically assessed using Student’s t-test for continuous variables and the chi-square (χ^2^) test for categorical variables, as appropriate.

The KAP and anticipatory action scores were categorized into three levels (low, moderate, and high) based on the Bloom’s cut-off point: <60% (low), 60–79% (moderate), and ≥80% (high) [31–32]. These categories were used exclusively for descriptive purposes. Due to low variability and the presence of categories with small or zero frequencies, the use of ordinal logistic regression was deemed inappropriate.

For inferential analysis, a count data modeling approach was adopted. A Poisson regression model was initially fitted. However, evidence of overdispersion was identified based on goodness-of-fit statistics and the ratio of the Pearson chi-square to its degrees of freedom. Additionally, the anticipatory action score had a mean of 9.69 (SD = 5.45), ranging from 0 to 27, with the variance (29.7) substantially exceeding the mean. These results indicated the presence of overdispersion in the data, therefore, a negative binomial regression model was used as a more appropriate alternative to the Poisson model (Additional file 3: Supplementary Tables S14-S17 and Supplementary Figure S2).

The fully adjusted model assessed the association between the gender variable and anticipatory action count scores, controlling for KAP domain scores and sociodemographic covariates. Results are presented as regression coefficients and incidence rate ratios (IRRs), with 95% confidence intervals. Robust standard errors were employed to address for potential violations of homoscedasticity. Statistical significance was set at the 5% level (p < 0.05).

## 3 RESULTS

### 3.1 Sociodemographic characteristics

Women were more likely to be widowed or single, while men were predominantly married or living in union (p < 0.001). Educational attainment did not differ significantly between genders (p = 0.264). Housing conditions also varied by gender. Women were more likely to live in houses with iron sheet roofs, whereas men more frequently reported roofs made of local materials (p < 0.001). The average household size (4 members) and monthly income (4,266 MZN) did not differ significantly between genders. However, men were significantly older on average than women (p = 0.004). Most households had soil/dung or cement floors, with no significant gender difference (p = 0.213) (Additional file 3: Supplementary Tables S6 and S7). These findings suggest important gender-related disparities in marital status, age, and housing conditions that may influence household vulnerability and preparedness in the context of cholera.

### 3.2 Awareness of cholera

Women reported greater awareness that cholera is associated with contaminated water (64% vs. 51%; p = 0.008) and unsanitary environments (23% vs. 15%; p = 0.032). This pattern suggests that women more frequently associate cholera with general environmental and household conditions. In contrast, men more frequently reported awareness that the disease can be transmitted through contaminated food or drinks (36% vs. 26%; p = 0.027), indicating gender differences in understanding of the specific transmission pathways. No statistically significant differences were observed between genders in overall awareness of cholera. Nearly all participants reported having heard of cholera (95.88% vs. 97.56%; p = 0.326), indicating a very high level of general awareness in both groups (Additional file 3: Supplementary Table S8).

Regarding sources of information, men reported greater exposure to media and technology as their main source of information about cholera (47% vs. 30%; p < 0.001). In contrast, access to information through the health system and healthcare professionals was similar between women and men (51% vs. 54%; p = 0.452), as was exposure to public health campaigns (54% vs. 45%; p = 0.073) (Additional file 3: Supplementary Table S8). This pattern may indicate greater exposure of men to media and digital communication channels, while the health system and public health campaigns appears to play a relatively similar role for both genders.

### 3.3 Attitudes toward cholera

Statistically significant differences were observed between women and men regarding trust in health authorities and the perception of stigma associated with cholera. Women demonstrated slightly higher trust in health authorities than men (96.9% vs 90.9%; p = 0.016), although the level of trust among those who reported trusting authorities was similar between genders (p = 0.918). Additionally, women more frequently reported the presence of stigma in the community (25.2% vs 16.0%; p = 0.019), particularly the perception of restrictions in access to public services (p = 0.043) (Additional file 3: Supplementary Table S9).

In contrast, no statistically significant gender differences were found in the perception of cholera severity (p = 0.246), perceived risk of infection (p = 0.911), overall perceived risk level (p = 0.164), community cooperation (p = 0.175), or perceived effectiveness of preventive measures, including handwashing, water treatment, food hygiene, and latrine use (p > 0.05) (Additional file 3: Supplementary Table S9). These findings indicate a generally similar perception between women and men across these aspects.

### 3.4 Hygiene and sanitation practices

Statistically significant gender differences were observed mainly in hygiene practices and in care-seeking behavior in cases of suspected cholera. Women more frequently reported washing their hands after using the latrine/toilet (87% vs 77%; p = 0.004), before preparing food (82% vs 72%; p = 0.011), and before meals (96% vs 89%; p = 0.004), suggesting greater adherence to preventive hygiene practices at critical points of transmission. In contrast, men more often reported rarely washing their hands with soap after defecation, compared with women (5.3% vs 0.9%; p = 0.032), indicating lower regularity in this preventive practice. In addition, men more frequently sought help from community health workers in cases of suspected cholera (17% vs 9%; p = 0.016). The remaining variables did not show statistically significant differences between genders (Additional file 3: Supplementary Table S10).

### 3.5 Gender-based distribution of KAP indicators

The composite analysis reveals a critical KAP gap characterized by low knowledge, relatively positive attitudes, and poor practices (Table 2). This pattern suggests that, although awareness of cholera may be high at the item level, it is not deeply rooted in knowledge and does not translate into effective preventive behaviors. In other words, providing information through awareness campaigns does not necessarily result in solid knowledge on the subject. In addition, willingness to act (attitude) does not necessarily translate into action, suggesting a gap between attitude and practice.

### 3.6 Preparedness before outbreaks

The existence of early warning systems for cholera outbreaks was the only variable that showed a statistically significant difference by gender, being more frequently reported by women compared to men (73.3% vs 61.5%; p = 0.008). This finding suggests greater women awareness or involvement in community surveillance and communication mechanisms related to cholera. The remaining variables did not show statistically significant differences. Among community preparedness actions for outbreaks, WASH-related activities and health education and awareness actions were highlighted, being reported similarly by both genders (p > 0.05). Regarding barriers to cholera preparedness, the main challenges identified were lack of financial resources (54%), limited access to clean water (41%), and insufficient information or awareness about the disease (34%), also with no significant differences between women and men (Additional file 3: Supplementary Table S11).

### 3.7 Actions during outbreaks

Statistically significant differences were mainly concentrated in immediate household responses and certain aspects of the community response to cholera outbreaks. Men more frequently reported contacting community health workers/APEs (26% vs 14%; p < 0.001), isolating symptomatic individuals at home (22% vs 12%; p = 0.004), avoiding gatherings (22% vs 15%; p = 0.037), and using mobile clinics or outreach teams during emergencies (14% vs 7%; p = 0.007) (Additional file 3: Supplementary Table S12).

In contrast, women more frequently reported increasing the consumption of safe or boiled water (36% vs 25%; p = 0.012), monitoring signs and symptoms among household members (56% vs 45%; p = 0.019), adopting measures recommended by local authorities or health professionals (55% vs 38%; p < 0.001), and safely storing rainwater (10% vs 5%; p = 0.046). Additionally, men more frequently identified coordination between local leaders and health authorities as part of the outbreak response (39% vs 29%; p = 0.049), suggesting greater awareness of the institutional role of community leadership. The remaining variables did not show statistically significant gender differences (Additional file 3: Supplementary Table S12).

Overall, these findings suggest that women tend to respond to outbreaks by increasing intra-household’s measures (domestic sphere) while men are more in contact with inter/external household’s actors (public domain) mimicking the stablished social norms.

### 3.8 Post-outbreak practices

After the end of the outbreak, no statistically significant gender differences were observed regarding the continuation of preventive measures adopted during the response phase (p = 0.432). In both groups, the majority of respondents reported fully maintaining these measures, with a slight predominance among women (66.7% vs 61.0%). However, there was a tendency toward greater partial maintenance among men (30.7% vs 25.5%), suggesting less consistent continuation of preventive practices in this group. A small proportion in both genders reported having completely discontinued the measures, with similar proportions (8.3% vs 7.8%) (Additional file 3: Supplementary Table S13).

### 3.9 Distribution of anticipatory actions (preparedness) levels by gender

Based on our methodology (the Bloom’s cut-off point), the vast majority of respondents were classified as having low preparedness levels, with almost all individuals falling into this category and an almost non-existent presence in the moderate and good categories. These results indicate a critically low level of anticipatory preparedness in the study population, suggesting a widespread lack of readiness. Gender differences were minimal, although a slightly higher proportion of men were classified at the moderate level compared to women, with no individuals recorded in the good preparedness category in either group (Table 3). This finding suggest that although people get awareness hardly they engage in implementing AA.

### 3.10 Negative binomial regression analysis

In the negative binomial regression analysis (Table 4), gender was not significantly associated with the number of AA (IRR = 0.993; 95% CI: 0.903–1.090; p = 0.882), suggesting that women and men tended to adopt a similar number of AA. Among the sociodemographic variables, the 36–60-year age group remained significantly associated with a higher expected number of AA compared with the 20–35-year age group (IRR = 1.107; 95% CI: 1.007–1.215; p = 0.035). Monthly income was also significantly associated with AA (IRR = 1.000; 95% CI: 1.000–1.000; p = 0.039). However, the point estimate and confidence interval of 1.000 indicate a negligible effect size, likely reflecting the influence of a small number of high-income outliers in a distribution characterized by extreme positive skewness.

Other sociodemographic variables, such as university education (IRR = 0.814; 95% CI: 0.645–1.028; p = 0.084) and household size (IRR = 1.018; 95% CI: 0.998–1.038; p = 0.073), showed marginal associations at the 10% significance level, however, these findings should be interpreted with caution, as the methodological criterion adopted in this study considers statistical significance at the 5% level. The remaining sociodemographic variables did not retain statistical significance in the adjusted model.

Regarding the KAP domains, knowledge and practices showed positive and statistically significant associations with the number of AA. Each one-unit increase in the knowledge score was associated with a 6.9% increase in the expected number of AAs (IRR = 1.069; 95% CI: 1.053–1.085; p < 0.001), while each one-unit increase in the practice score was associated with a 7.2% increase (IRR = 1.072; 95% CI: 1.054–1.090; p < 0.001). In contrast, the attitude score was not significantly associated with AA (IRR = 1.008; 95% CI: 0.982–1.034; p = 0.549).

Overall, the results suggest that the influence of sociodemographic characteristics on AA does not occur directly, but predominantly through levels of knowledge and the adoption of practices. In substantive terms, individuals with greater knowledge and better practices tended to report a higher number of AA, regardless of their sociodemographic characteristics. These findings indicate that the adoption of AA fundamentally depends on the ability to translate knowledge into effective behavior, reinforcing the importance of interventions focused on strengthening knowledge and promoting concrete practices.

## 4` Discussion

The results highlight marked sociodemographic inequalities conditions, consistent with previous findings [33–34]. In this study, women were, on average, younger, more frequently widowed. Although women also exhibited relatively lower educational attainment than men, this difference was not statistically significant. This pattern reflects structural dynamics commonly observed in rural contexts in Mozambique, where access to formal education remains unequal between men and women [35]. Consequently, lower levels of education among women suggest lower health literacy [36], which may limit their ability to adequately understand and respond to risks such as cholera.

Despite these structural limitations, women reported greater adoption of preventive practices, such as water treatment and handwashing. This finding may be explained by the social roles traditionally assigned to women, who often assume responsibility for water collection and household hygiene [37]. Thus, even in resource-constrained settings, their greater involvement in household health-related activities may facilitate the adoption of protective behaviors.

With regard to knowledge of cholera, an important gap was observed: although men and women recognized basic causes of cholera, such as contaminated water and poor hygiene conditions, a more in-depth understanding of transmission pathways and symptoms remained limited. This pattern, consistent with contexts of generally low educational attainment, raises concerns, as incomplete knowledge may hinder the adoption of effective responses. Even so, gender differences emerged in how risk is interpreted: while women tended to emphasize individual preventive practices, men more frequently referred to structural factors, such as the lack of sanitation. This pattern partially contrasts with previous findings, which associated women with greater knowledge and more favorable attitudes toward WASH [14]. Thus, although both studies identify a gender effect, the nature of the differences diverges, possibly due to contextual variation, methodological differences, and exposure to local interventions.

With regard to attitudes toward cholera, a high perception of cholera severity and strong trust in health authorities were observed, with women demonstrating high trust in health authorities. However, this positive attitude was not fully reflected in practices, which remained generally suboptimal. Similar findings were reported [19,38], reinforcing the idea that knowledge and attitudes alone are not sufficient to ensure consistent preventive behaviors.

In this context, structural factors play a decisive role. Limited access to safe drinking water and adequate sanitation, widely reported in this study without statistically significant gender differences, represents a concrete barrier to the adoption of preventive measures. Improvements in WASH infrastructure have a direct impact, particularly for women, by reducing the burden of domestic work and facilitating hygiene practices [37]. Thus, the persistence of inadequate practices appears to reflect less an individual failure and more a set of material and structural constraints that limit the translation of knowledge and attitudes into effective action. In short, this may be interpreted as meaning that people may have knowledge and positive attitudes, but lack the resources to act.

The critically low overall level of AA preparedness, with no significant gender differences in the final Bloom-based classification, is consistent with the recent literature on AA, which emphasizes the fundamental role of structured anticipatory systems based on predefined triggers, early warning mechanisms, and operational plans capable of mobilizing resources before epidemic peaks [10]. In the absence of such an architecture, responses tend to remain predominantly reactive and dependent on local structures that are often overburdened [10]. In this sense, the low level of preparedness observed suggests not only community-level limitations, but also weaknesses in anticipatory response capacity.

Additionally, evidence on cholera response indicates that localized and rapid interventions, such as case-area targeted interventions, depend heavily on community coordination, trust in health facilities, and the availability of WASH services [39], factors that proved fragile in this study except for trust in health facilities. Finally, the gender differences observed in risk perception are consistent with the literature on protective behaviors, which suggests that women tend to have higher risk perception and greater adherence to preventive measures than men [40]. This pattern helps explain greater women community engagement, while underestimation of risk among some men may undermine the effectiveness of collective preparedness strategies.

In the multivariable negative binomial regression, gender was not significantly associated with AA, suggesting similar levels of adoption of AA among women and men. Among the sociodemographic variables, the 36–60 age group remained significantly associated with a 10.6% increase in the expected number of AA compared with the 20–35 age group. While monthly income reached statistical significance (p = 0.039), the IRR of 1.000 indicates a negligible effect size, likely attributable to the measurement of income in small nominal units (MZN) rather than a meaningful dose-response relationship. The practical relevance of this finding is therefore limited and should be interpreted cautiously.

Regarding the KAP domains, knowledge and practices remained significantly associated with AA, with each one-unit increase in the respective scores corresponding to approximately 6.9% and 7.2% increases in the expected number of AA, respectively, whereas attitudes were not significantly associated. Overall, these findings suggest that preparedness appears to be more strongly determined by operational preventive capacities than by sociodemographic characteristics alone.

KAP studies in contexts such as Kenya have shown that high levels of knowledge do not necessarily translate into appropriate practices, particularly in settings characterized by material constraints such as limited access to treated water [19]. In contrast, evidence from contexts such as Lebanon suggests a positive association between knowledge and practices, indicating that this relationship is highly dependent on contextual factors, including infrastructure, institutional support, and socioeconomic conditions [17]. Thus, the findings of this study may reflect not only the effect of knowledge itself, but also its interaction with conditions that enable its translation into practice.

The absence of a statistically significant direct effect of gender differences in the adjusted model should not be construed as evidence that gender inequalities are absent. Rather, gender may operate through indirect pathways, potentially mediated by education, KAP levels, and differential access to WASH services, though these mediating mechanisms were not formally tested in the present study and therefore remain theoretical. In this sense, the patterns observed in this study suggest that AA emerge from the intersection of individual capacities, social norms, and structural conditions. Consequently, interventions focused exclusively on information dissemination are likely to have limited impact unless they are accompanied by investments that enable the practical adoption of these measures, including improvements in WASH infrastructure and strategies that account for gender differences in risk perception and response [10,23,41].

## 5 Conclusions

The results show that cholera preparedness in Zambézia Province remains critically limited, with most participants exhibiting low levels of AA. The findings reveal an important gap between awareness of the disease, effective knowledge, and the implementation of preventive behaviors. Although most respondents demonstrated high awareness of cholera and generally positive attitudes toward the disease, these aspects did not necessarily translate into appropriate practices or greater preparedness. The multivariable analysis showed that knowledge and practices were the main factors associated with the adoption of AA, whereas attitudes were not significantly associated. Among the sociodemographic variables, only the 36–60 age group remained significantly associated with preparedness. Gender was not a significant independent predictor of AA in the adjusted model, suggesting that any gender-related influence on preparedness may operate through indirect mechanisms rather than as a direct structural determinant.

From public policy perspective, the results underscore the need for integrated interventions that combine health education, improvements in WASH conditions, community-based early warning mechanisms, and gender-sensitive strategies. Priority should be given to strengthening accurate, actionable knowledge and promoting sustainable preventive practices, as these were the two factors most strongly associated with AA adoption in this study. Such efforts could help transform disease awareness into effective preparedness and reduce reliance on reactive responses to cholera outbreaks.

## 6 Limitations

The study has some limitations. The sampling strategy prioritized the most affected neighborhoods, which may have limited the representativeness of the findings for less affected areas. In addition, the absence of qualitative data constitutes another limitation, as such information could have contributed to a more in-depth and contextualized interpretation of the quantitative findings. Nevertheless, the findings provide important evidence on KAP and anticipatory actions in highly vulnerable settings and should be interpreted within the context of the study area.

### Supplementary Material

Supplementary Files

This is a list of supplementary files associated with this preprint. Click to download.

- Additionalfile1.Figure1Conceptualframeworkofthestudy.png
- Additionalfile2.Figure2Studyareamap.pdf
- Additionalfile3.SupplementaryInformation.docx
- Additionalfile4.DescriptiveStatisticalAnalysis.do
- Additionalfile5.KAPandAAScoreClassificationUsingBloomsCutoff.do
- Additionalfile6.NegativeBinomialRegressionandModelDiagnostics.do
- Additionalfile7.DeidentifiedAnalyticalDataset.dta

## Figures and Tables

**Fig. 1:**
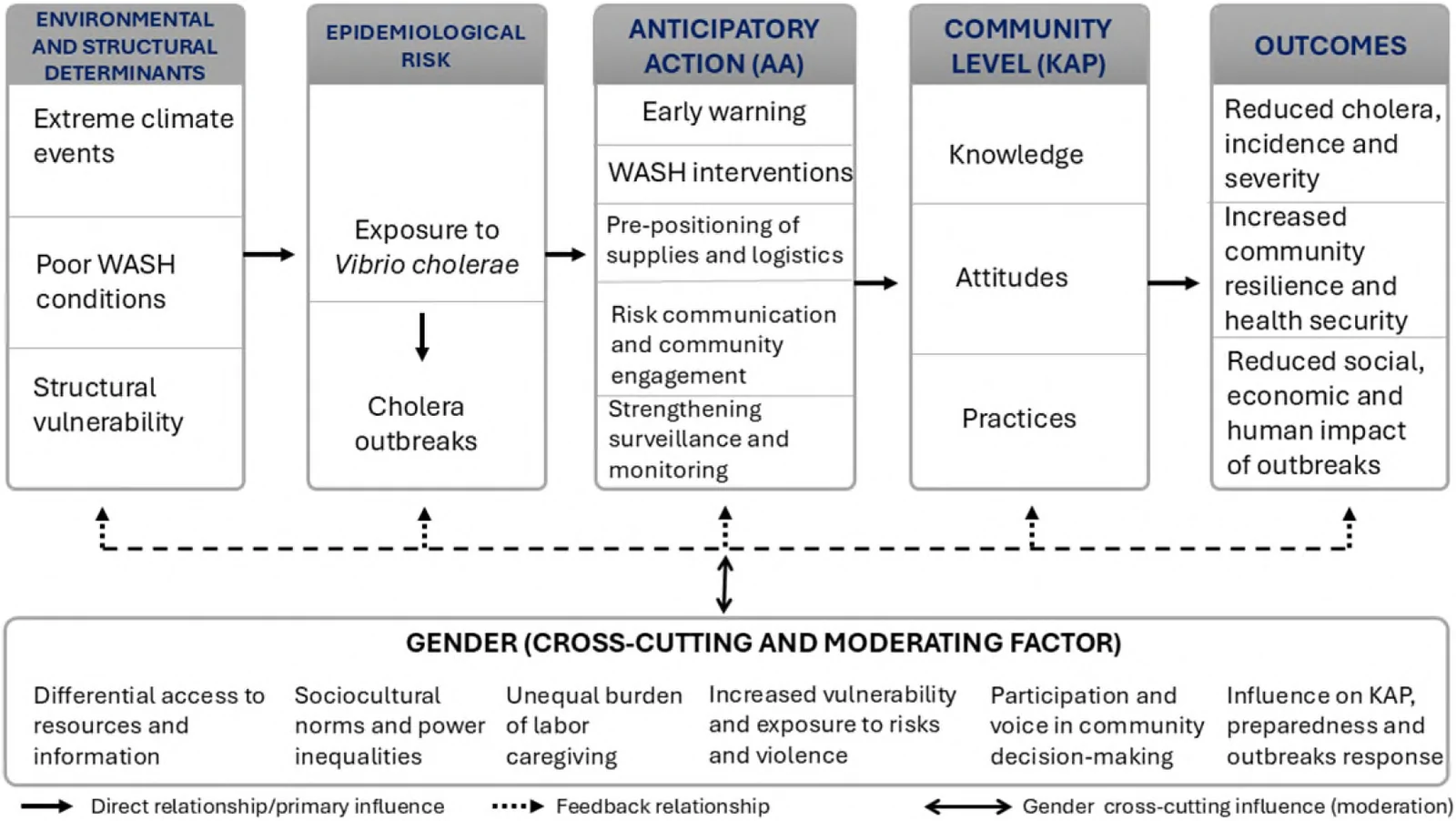
Conceptual framework of AA, KAP, cholera risk, and gender Source: Author’s own elaboration based on literature review

**Fig. 2:**
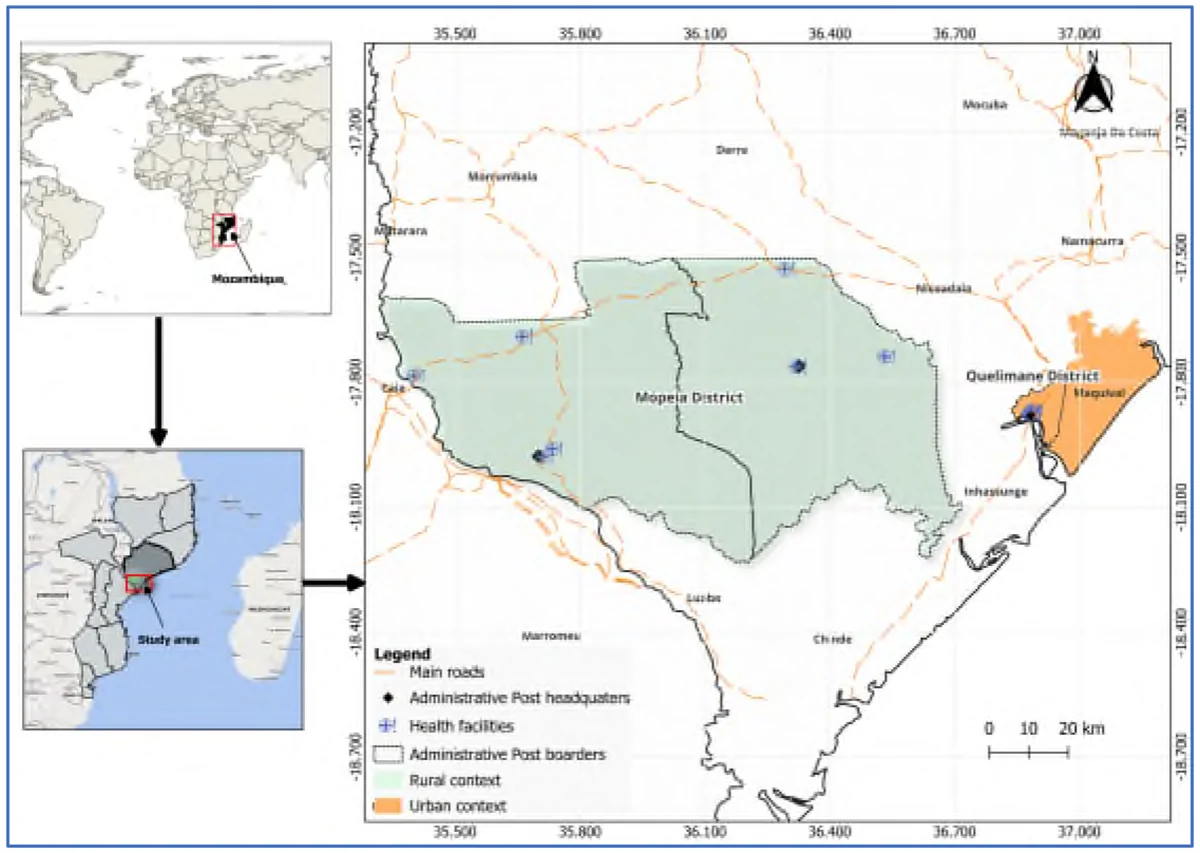
Research site Source: Authors

**Table 1:** Gender distribution of respondents across districts

Gender	District		Total
	Quelimane	Mopeia	
	N	N	N
Women	152	91	243
Men	87	118	205
**Total**	239	209	448

Note: N – Frequencies.

**Table 2:** Gender distribution of KAP levels related to cholera.

Domains		Gender					
	Category	Women		Men		Total	
		N	%	N	%	N	%
knowledge	Poor	235.00	96.71	196.00	95.61	431.00	96.21
	Moderate	6.00	2.47	9.00	4.39	15.00	3.35
	Good	2.00	0.82	0.00	0.00	2.00	0.45
Attitude	Poor	31.00	12.76	23.00	11.22	54.00	12.05
	Moderate	28.00	11.52	24.00	11.71	52.00	11.61
	Good	184.00	75.72	158.00	77.07	342.00	76.34
Practice	Poor	206.00	84.77	182.00	88.78	388.00	86.61
	Moderate	37.00	15.23	23.00	11.22	60.00	13.39
	Good	0.00	0.00	0.00	0.00	0.00	0.00

Note: N – Frequencies; % - Percentages.

**Table 3:** Gender distribution of anticipatory action (preparedness) levels.

		Gender					
Variable	Category	Women		Men		Total	
		N	%	N	%	N	%
Anticipatory actions / Preparedness	Poor	234.00	96.30	200.00	97.56	434.00	96.88
	Moderate	9.00	3.70	5.00	2.44	14.00	3.13
	Good	0.00	0.00	0.00	0.00	0.00	0.00

Note: N – Frequencies; % - Percentages.

**Table 4:** Multivariable negative binomial regression results for factors associated with AA.

Variables	β (SE)	95% CI (β)	ρ-value	IRR	95% CI (IRR)
**Gender**					
Women	Ref.			Ref.	
Men	−0.007 (0.047)	−0.100 to 0.086	0.882	0.993	0.903 to 1.090
**Age**					
20–35	Ref.			Ref.	
36–60	0.101 (0.047)	0.007 to 0.195	0.035 **	1.107	1.007 to 1.215
>60	−0.016 (0.074)	−0.129 to 0.162	0.825	1.017	0.878 to 1.176
**Marital status**					
Single/never married	Ref.			Ref.	
Union/married	−0.121 (0.077)	−0.272 to 0.029	0.116	0.886	0.761 to 1.030
Divorced/separated	0.071 (0.158)	−0.239 to 0.381	0.653	1.074	0.787 to 1.464
Widow/widower	−0.086 (0.097)	−0.278 to 0.105	0.378	0.917	0.757 to 1.111
**Educational level**					
No formal education	Ref.			Ref.	
Primary	0.039 (0.064)	−0.086 to 0.165	0.538	1.040	0.917 to 1.179
Secondary	0.036 (0.069)	−0.098 to 0.171	0.596	1.037	0.906 to 1.187
University	−0.205 (0.118)	−0.438 to 0.027	0.084*	0.814	0.645 to 1.028
**Household size**	0.017 (0.010)	−0.001 to 0.037	0.073*	1.018	0.998 to 1.038
**Monthly income**	0.000 (0.000)	0.000 to 0.000	0.039**	1.000	1 to 1.000
**Climate-related**					
**displacement**	−0.039 (0.041)	−0.120 to 0.041	0.344	0.962	0.886 to 1.042
**Knowledge score**	0.066 (0.007)	0.053 to 0.086	0.000***	1.069	1.053 to 1.085
**Attitude score**	0.007 (0.013)	−0.017 to 0.033	0.549	1.008	0.982 to 1.034
**Practice score**	0.069 (0.008)	0.053 to 0.086	0.000***	1.072	1.054 to 1.090
		N =	448		
		Wald chi2 =	314.85		
		Prob > chi2 =	0.0000		

Note: N= number of observations; β = regression coefficient; SE = standard error; IRR = incidence rate ratio; 95% CI = 95% confidence interval; IRR values > 1 indicate an increase in the expected number of anticipatory actions, whereas values < 1 indicate a decrease.

Statistical significance levels are indicated by *** p < 0.01, ** p < 0.05, and * p < 0.10.

## Data Availability

The raw data analyzed during the current study are available as a supplementary file.

## Abbreviations

KAP: Knowledge, Attitudes, and Practices
AA: Anticipatory actions
WBD: Waterborne diseases
WASH: Water, Sanitation and Hygiene
IRR: Incidence Rate Ratio
CI: Confidence interval
